# Impact of Endocrine Disruptors on the Genitourinary Tract

**DOI:** 10.3390/jox14040099

**Published:** 2024-12-02

**Authors:** Christophe Caneparo, Laurence Carignan, Elena Lonina, Sarah-Maude Goulet, Felix-Antoine Pellerin, Stéphane Chabaud, François Bordeleau, Stéphane Bolduc, Martin Pelletier

**Affiliations:** 1 Department of Pediatrics, Gynecology and Obstetrics, Faculty of Medicine, Geneva University Hospitals, University of Geneva, CH-1205 Geneva, Switzerland; 2 Oncology Division, CHU de Québec-Université Laval Research Center and Université Laval Cancer Research Center, Quebec, QC G1R 3S3, Canadafelix-antoine.pellerin@crchudequebec.ulaval.ca (F.-A.P.); francois.bordeleau@crchudequebec.ulaval.ca (F.B.); 3 Regenerative Medicine Division, Centre de Recherche en Organogénèse Expérimentale/LOEX, CHU de Québec-Université Laval Research Center, Université Laval, Quebec, QC G1J 5B3, Canada; stephane.chabaud@crchudequebec.ulaval.ca (S.C.); stephane.bolduc@fmed.ulaval.ca (S.B.); 4 Infectious and Immune Diseases Division, CHU de Québec-Université Laval Research Center, Quebec, QC G1V 4G2, Canada; 5 Intersectorial Centre for Endocrine Disruptors Analysis, Institut National de La Recherche Scientifique (INRS), Montreal, QC H4V 1B7, Canada; 6 Department of Microbiology-Infectious Diseases and Immunology, Faculty of Medicine, Laval University; ARThrite Research Center, Université Laval, Quebec, QC G1V 0A6, Canada; 7 Department of Molecular Biology, Medical Biochemistry and Pathology, Université Laval, Quebec, QC G1V 0A6, Canada; 8 Department of Surgery, Université Laval, Quebec, QC G1V 0A6, Canada

**Keywords:** endocrine disruptor, hormone, genitourinary tract, bladder, urethra, prostate, testicles, uterus, cervix, ovary

## Abstract

Over the last decades, the human species has seen an increase in the incidence of pathologies linked to the genitourinary tract. Observations in animals have allowed us to link these increases, at least in part, to changes in the environment and, in particular, to an increasing presence of endocrine disruptors. These can be physical agents, such as light or heat; natural products, such as phytoestrogens; or chemicals produced by humans. Endocrine disruptors may interfere with the signaling pathways mediated by the endocrine system, particularly those linked to sex hormones. These factors and their general effects are presented before focusing on the male and female genitourinary tracts by describing their anatomy, development, and pathologies, including bladder and prostate cancer.

## 1. Introduction

Living species, including humans, interact closely with their environment. While some species can use objects as tools, humans have mechanized and manipulated their environments in unique ways. The Industrial Revolution accelerated this domestication, leading to changes in production, plastic use, and increased exposure to pesticides, some of which have demonstrated their toxicity to humans [1]. Most of these products are now known as endocrine-disrupting chemicals (EDCs) due to their effect on the endocrine system. In many cases, new products that are used daily contain chemicals whose effects on our species have been poorly evaluated [2,3,4,5]. For example, toxicity tests are mainly based on acute exposure, whereas these chemicals are chronically present in our environment. Furthermore, chemicals are usually evaluated individually, while a combination of dozens exists in the environment [6,7]. In addition, the potentially harmful effect of these components may combine with those linked to the lifestyle of more urban, more sedentary, and older populations [8,9].

Humans living in rural areas are exposed to various environmental stressors, such as pesticides, from their use in agriculture. Yet, people living in urban areas also face various environmental stressors, from road pollution to industrial emissions and household pollutants [10]. These stress factors can weaken individuals, particularly when combined with a sedentary lifestyle resulting from mechanization. Over time, this lifestyle can induce obesogenic behaviors likely to exacerbate the effects of EDCs, mainly because of the critical role of adipose tissues in endocrine control [11,12,13]. The improvements in medicine, hygiene, and overall living conditions, mainly during the 20th century, allowed a notable increase in life expectancy, increasing the number of older people and generating new medical challenges [14].

Over the years, increasing evidence has arisen on the adverse effects of EDCs on human fertility, including a decrease in sperm counts and quality that has been observed in both developed and developing countries [15,16]. Although this phenomenon can be attributed to causes such as obesity and lifestyle, studies have linked infertility to exposure to pollutants, including endocrine disruptors [16]. However, conflicting results and cultural biases make it difficult to reach a definitive consensus [17,18]. Furthermore, the assessment of reproductive capacity must consider not only cellular or tissue perspectives but also behavioral, social, and cultural factors, including their influence on sexual conduct [19] and mate choice [20].

Scientists are increasingly realizing that disease mechanisms in complex organisms can result from multiple and multidisciplinary, even interdisciplinary, causes, highlighting the need to improve the approaches to address these challenges [21]. The present review describes different EDCs and their mechanisms of action, connecting their effects on the endocrine system and the development of pathologies associated with genitourinary organs, ranging from their impact on immune cells to cancers. Finally, we investigate the state of regulations of EDCs, highlighting the importance of better controlling their use and release into the environment.

## 2. Endocrine Disruptors

Hormones are endogenous molecules synthesized in the body in small amounts and released at specific times and locations to regulate crucial biological processes such as development, metabolism, immunity, and reproduction [19,22]. Endocrine disruptors are physical or chemical agents able to alter either the expression, secretion, transport, binding, or activity of endogenous endocrine hormones and mimic their actions [7]. In 2002, the World Health Organization defined EDCs as “exogenous substances or mixtures that alter the function(s) of the endocrine system and consequently cause adverse effects in an intact organism, its progeny, or its (sub)populations” [1,19,23]. The European Union’s registration, evaluation, authorization, and restriction of chemicals regulation reports that 1000 chemicals out of approximately 140,000 are considered EDCs [10]. Nonetheless, the United States produces or imports more than 10 tons of EDCs per person per year [24], highlighting the prevalence of these products in our daily lives despite their recognition as potentially harmful products.

**Figure 1:**
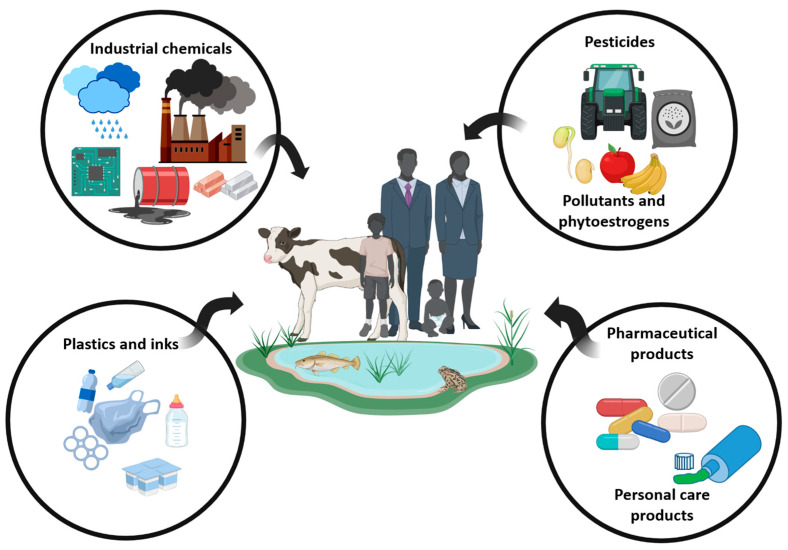
**Sources of EDC contamination.** EDCs from various origins, such as industrial chemicals, can directly contaminate soil and water by spilling or as rain carries the contaminants in the fumes. Heavy metals are also present in many industrial processes. Plastics and inks used for receipts can also contaminate food or the environment when they become garbage. Pesticides are widely used in agriculture and can contaminate soil, water sources, and produce. Natural EDCs, such as phytoestrogens, can also be present in certain products like soya. Finally, pharmaceutical and cosmetic/personal care industries use many EDCs in various production processes. All these contaminants can negatively affect the health of humans and animals, especially those in direct contact with polluted water, like fish and amphibians.

Endocrine disruptors can be divided into two main categories: physical, such as temperature and light, or chemical, including synthetic and natural endocrine disruptors, such as phytoestrogen [25]. Most EDCs have a similar structure to endogenous hormones and can activate or inhibit their signaling pathway, leading to dramatic consequences in various organ systems, including the reproductive system, which is essential to the survival of a species [25,26,27]. EDCs are being constantly produced and introduced into the environment, causing continuous exposure to humans and wildlife as they are found in soil, dust, and water [28]. The widespread use of EDCs in our environment (Figure 1) led to a warning statement by the Endocrine Society, namely that EDCs are a significant concern for public health [29,30]. This concern became particularly true after discovering the transgenerational persistence of the effects of EDCs through epigenetic modifications both in men and women [31,32].

Some studies suggest human exposure to natural endocrine disruptors, which often have higher or comparable endocrine activity than synthetic ones, has been substantial over time [33]. As a result, potential adaptation mechanisms to these natural compounds may have evolved, with some adaptations possibly arising more recently due to factors like dietary changes. It is, therefore, crucial to assess the distinct effects of both natural and synthetic endocrine disruptors to better understand how they may each negatively impact human physiology.

### 2.1. Classification of Endocrine-Disrupting Chemicals

Endocrine-disrupting chemicals can be classified into physical, natural, and synthetic, depending on their source and overall composition. A summary of this classification and examples can be found in Table 1, which also includes the molecular structures of natural hormones for comparison.

#### 2.1.1. Physical Endocrine Disruptors

The physical environment of the human species has remained stable for most of its existence, with alternating day/night cycles and temperatures varying with the seasons [34]. Animal species generally synchronize their hormonal rhythms to adapt to their environment [35]. Changes in lifestyle, such as urbanization, the use of screens, and night work expose humans to artificial sources of light that affect biological processes and induce endocrine alterations. For instance, human exposure to artificial light has been linked to an increased incidence of certain cancers and obesity, which influences human health and reproductive functions [36].

Temperature can play various roles in biological processes, such as in the determination of sex during embryonal development, and changes in these environmental conditions can alter proper development. For instance, the increase in global temperatures has been suggested to change the sexual development of certain vertebrate animals, such as frogs [37]. The effects of increased temperatures have been further demonstrated in more complex animals, such as dairy cows, where the maturation of oocytes and the development of embryos can be affected, ultimately impacting the fertility of these animals [38]. Although there is no direct link between increasing temperatures affecting the development of the body and the increasing incidence of reproductive pathologies, temperature has been shown to play a potential role in exacerbating the effects of other EDCs [37], increasing the complexity of these phenomena.

#### 2.1.2. Natural Endocrine Disruptors

Phytoestrogens are naturally occurring, non-steroidal polyphenols sharing structure similarity to estradiol [39,40]. Six classes of phytoestrogens have been defined: flavonoids/isoflavonoids, pterocarpans, enterolignans, coumestans, mycotoxins (such as zearalenone), and stilbenes [41]. Humans are exposed to phytoestrogens through their food [42]. Phytoestrogens can have potential roles in human health, including menopausal symptoms such as hormonal cancers, osteoporosis, and cardiovascular diseases [39]. However, besides these adverse effects, flavonoids (such as genistein, S-equol, and naringenin) and stilbenes (such as resveratrol and pterostilbene) have been used in several clinical trials to treat breast cancer [43]. Phytoestrogens have also shown a beneficial role in the treatment of Alzheimer’s disease [44]. Interestingly, a study tried to explain the Greek myth of Hermaphroditus, i.e., the presence of both male and female characteristics in one individual, by an exposition to natural endocrine disruptors such as zearalenone after bathing in Salmacis Lake [45].

#### 2.1.3. Synthetic Endocrine Disruptors or Endocrine-Disrupting Chemicals

Several hundred synthetic molecules in our environment have been identified as potential EDCs by modulating endogenous hormones’ action and disrupting multiple cellular and tissue functions [10].

Several molecules, such as phthalates and bisphenols, are useful in plastic synthesis. Phthalates (phthalic acid esters) are compounds used for the synthesis of polyvinyl chloride (PVC), cosmetics, and medical products [32,46]. Consequently, due to their high human exposure, phthalates and bisphenols have attracted a lot of attention in the last decades. These estrogenic monomers are used to produce polycarbonate plastics, epoxy resins, and some industrial coatings, such as thermal receipts [25,32]. Following increased public awareness, several “BPA-free” products have appeared in the market, but the identity of compound(s) used to replace BPA and their potential toxicity remains largely unknown. For example, the testicular toxicity of bisphenol AF (BPAF) and bisphenol S (BPS) is roughly comparable to that of bisphenol A (BPA) [47]. Similarly, bisphenol-free plastics containing the substitute Tritan have also been shown to leach chemicals with estrogenic activities [48].

Other EDCs commonly present are pesticides used in herbicides, insecticides, fungicides, and rodenticides. Pesticides include dichlorodiphenyltrichloroethane (DDT), dichlorodiphenoxy-dichloroethylene (DDE), trazine, methoxychlo, and vinclozolin [32]. Moreover, chemicals such as diethylstilbestrol (DES) have been extensively used in pharmaceutical drugs before their effects were detected [7]. Parabens, such as methylparaben, butylparaben, and benzylparaben, are also used as anti-microbial agents in food, pharmaceutics, and cosmetics [49].

Some EDCs are also considered persistent environmental contaminants for their long lifetime [50,51]. For example, polychlorinated biphenyls (PCB) are used in plasticizers, carbonless copy paper, wax extender ink, hydraulic fluid, and lubricants [52]. Dioxins, like polychlorodibenzo-p-dioxine (PCDD), are also in this group of products. They are produced from various manufacturing activities, and they have demonstrated high acute toxicity and chronic effects in both animals and humans [53,54]. Similarly, brominated flame retardants, such as polybrominated diphenyl ethers (PBDE), are also widely used. Although they are banned from several countries [55], they can still be found in everyday products [56].

Another category of EDCs is heavy metals like the ones found in cigarette smoke (e.g., chromium, manganese, nickel, copper, zinc, arsenic, barium, cadmium, mercury, and lead). Their effects on human health are generally well documented, even if their EDC potential was often neglected [57].

### 2.2. Mechanisms of Action of EDCs

**Figure 2:**
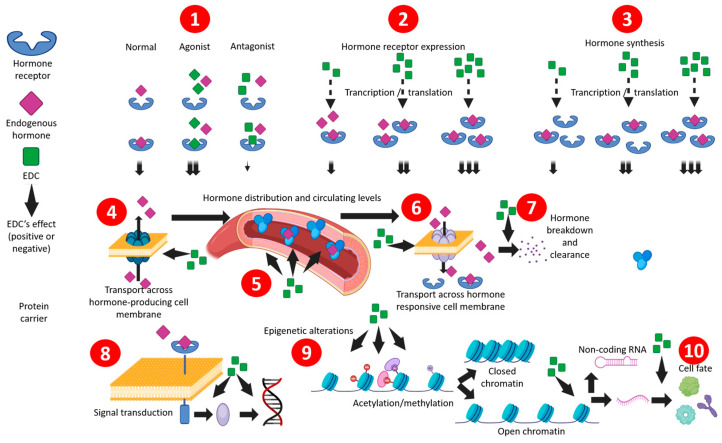
**Mechanisms of action of EDCs.** EDCs can interact through various mechanisms, such as hormonal receptors. These interactions can lead to dysregulated hormonal levels, availabilities, and distribution, ultimately impairing the actions of endogenous hormones in their target tissues. Furthermore, some EDCs possess off-target effects and may interact directly with cells and the genome. The ten different mechanisms of action are detailed in Section 2.2. This figure was inspired by the review from La Merrill et al. [59].

Various EDCs can cause adverse effects in physiological or pathological biological phenomena such as development, immune response, cell proliferation and differentiation, and cancer [58]. Recently, key characteristics of EDCs have been identified, as shown in Figure 2 [59]. EDCs can (1) bind/interact with hormone receptors and serve as agonists [60] or antagonists [61,62], increasing or decreasing cell signaling. EDCs can (2) modulate transcriptional or translational events; modify the expression, localization, and internalization of hormone receptors [63,64]; as well as (3) alter hormone synthesis [65,66]. 4) EDCs can further (4) interfere with the secretion of hormones [60], (5) affecting their distribution and circulation levels [67], as well as (6) the transport of the hormone in the target cells. These chemicals can also (7) interfere with the breakdown and clearance of hormones, potentially increasing their presence and effects [68]. EDCs can also (8) attenuate or potentiate the signal transduction inside hormone-responsive cells, modulating the activator or repressor of expression, including the signaling cascade to finally modulate the downstream targets. More subtly but often permanent, EDCs can also have (9) epigenetic effects that can render genes more or less accessible through methylation and acetylation to close or open the chromatin, further modulating the expression of non-coding RNA, which will act on their targets [69,70]. Finally, (10) cell fate can also be manipulated by EDCs to induce inappropriate proliferation, differentiation, and apoptosis, especially during the organism’s development [71,72].

### 2.3. Interplay with Normal Cell Estrogenic Signaling

Estrogens are female hormones that play essential roles in several biological processes. Like androgens, estrogens are part of the family of steroid compounds. They mainly include estrone, estradiol, estriol, and estetrol [73]. Estrogens are primarily synthesized in the ovaries but also by the adrenal glands and adipose tissues [73]. EDCs’ action and the modification of hormonal balance may contribute to obesity [11,12]. Estradiol is the primary circulating form of estrogens, and it is synthesized from cholesterol from low-density lipoproteins (LDL) by the granulosa cells of the ovarian follicles and the corpora lutea using aromatases [74]. Although androgens are the predominant male hormones [75], they can also be produced in females, and their imbalance in both males and females can induce pathologies [23]. It can be noted that, depending on the concentration, estrogen mimetic molecules can also activate the androgen receptor (AR), such as seen with BPA [23].

In addition to their reproductive functions, such as regulating the menstrual cycle, estrogens control bone density, brain function, and inflammation regulation [76]. Estrogens exert their effects through several signaling pathways, including binding to their cytoplasmic estrogen receptors (Erα and Erβ), which induce their translocation into the cell nucleus and binding to estrogen response elements (EREs), thus acting as transcription factors [77]. Another estrogen receptor has been recently described: the G protein-coupled estrogen receptor (GPER), a membrane-associated receptor [78,79]. Estrogen signaling can further lead to transcriptional complexes targeting non-ERE sequences and exert non-genomic effects [73].

EDCs presenting structural similarities to estrogens can signal through the same pathways by binding to the hormones or competitively binding to receptors, mimicking estrogenic actions or causing inadequate receptor signaling [80]. Interestingly, some metal ions, known as metalloestrogens, can further exert estrogen activity (aluminum, antimony, barium, cadmium, chromium, cobalt, copper, lead, mercury, nickel, arsenite, selenite, and vanadate) [81]. EDCs can also exert their effects through progesterone, thyroid, or retinoid receptors [49]. Nonetheless, other mechanisms involving different signaling pathways could be used depending on the EDC, the concentration, and the target cell.

## 3. Goods and Bads: The Effects of EDCs on Human Health

**Figure 3:**
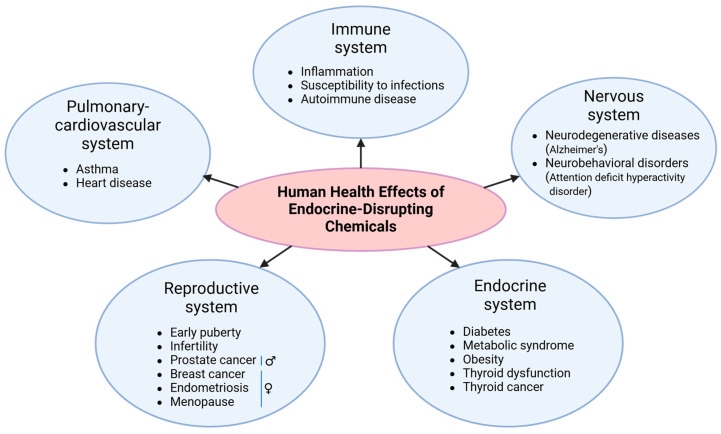
**Multisystem effects of EDCs on human health.** Various EDCs, such as bisphenols, phthalates, pesticides, dioxin, and phytoestrogens, can impact human biological systems, including endocrine, nervous, immune, pulmonary-cardiovascular, and reproductive systems. These effects vary widely and have significant implications for overall human health.

Some hormone treatments, such as selective estrogen receptor modulators (SERMs), act as estrogen agonists or antagonists, classifying them as EDCs by definition [82]. The use of these SERMs for treating conditions such as breast cancer or osteoporosis [83], as well as for hormone therapy in sexual reassignment [84] or in vitro fertilization [85,86], is increasing in response to rising demand from the pharmaceutical market [29]. Several EDCs are also present in the environment and everyday consumer products, mimicking, blocking, or interfering with the body’s natural hormones, leading to developmental and biological effects [5,87,88]. EDCs are mainly known for their adverse effects on humans. Numerous studies have demonstrated that these chemicals can be detected in bodily fluids and have functional impacts [49,89,90,91,92,93,94,95]. Examples include bisphenols, phthalates, and pesticides, which could affect endocrine, immune, and neurological systems and disrupt hormonal balance. Some EDCs have also been linked to tumor growth (Figure 3) [87,96,97,98,99,100,101,102,103,104,105,106,107]. The kidneys are primary organs in the urinary system and are a major site for accumulating exogenous substances like EDCs. Studies have shown that EDCs can cause urinary issues such as proteinuria and can impair the function of the glomeruli and renal tubules [108,109]. As these chemicals can affect hormone-sensitive organs and other systems, such as the immune system, and potentially contribute to cancer development, it is, therefore, crucial to understand the mechanisms of action of EDCs to minimize their impact on human health.

### Effect of EDCs on the Immune System

Often overlooked, immune cells play crucial roles in maintaining homeostasis and encouraging the proper functioning of the genitourinary organs. When looking into the female reproductive system, various immune cells, including dendritic cells (DCs), macrophages, regulatory T cells (Tregs), and neutrophils, are implicated in the menstruation cycle as they promote tissue remodeling while preventing excessive inflammation [110]. These cells also play roles in clearing potential infections in the vagina after coitus to promote a favorable environment for insemination [111,112]. Moreover, immune cells are involved in early and ongoing pregnancy. Modulated by steroid hormones and seminal fluid, Tregs expand to promote tolerance and avoid overactivation of effector cells against fetal antigens, which may lead to poor pregnancy outcomes [113]. Tregs, macrophages, mast cells, and NK cells also play roles in vascular and tissue remodeling, contributing to blastocyst implantation and healthy pregnancies [114,115,116,117,118].

The male reproductive system is similarly abundant in immune cells. The penis and urethra are considered classical mucosal sites where macrophages and NK cells are the first responders to infection [119]. Memory B cells and plasma cells also contribute to protective immunity by secreting IgG and IgA, while resting memory T cells orchestrate immune responses that might arise [119]. The testis is considered an immune-privileged organ as it must limit antigenic presentation to avoid immune over-activation against spermatogenic cells that could compromise sperm quality [120]. Nonetheless, various immune cells like macrophages, DCs, NK cells, mast cells, and T cells reside in the interstitium [120]. Moreover, Sertoli and Leydig cells in the testes express various pattern-recognition receptors. They can initiate inflammation by releasing multiple cytokines that recruit neutrophils, macrophages, and T cells during testicular infection [120].

Higher in the urinary tract, urothelial cells are the first barrier against pathogens. They renew themselves continuously and secrete various anti-microbial peptides [121]. Upon infection, urothelial cells and resident mast cells secrete inflammatory mediators, including cytokines and histamine, to recruit other immune cells [121]. Mast cells also release IL-10, which promotes urothelial exfoliation and tissue repair while curling inflammation by encouraging Th2 polarization in the kidneys and bladder [121,122,123]. Similarly, neutrophils participate in bacterial clearance by releasing reactive oxygen species, cytokines, and anti-microbial peptides and in the modulation of inflammation by limiting tissue damage [123]. Finally, macrophages are abundant immune cells that reside in the urinary organs. They can define the course of infection depending on their polarization and by mediating the interplay between other immune cells [124]. Undeniably, our knowledge of the diversity of immune cells in the genitourinary system has increased, but their importance in reproductive and urinary health remains elusive.

Extensive literature has highlighted the vast effects of EDCs on reproductive health, kidney injury, and comorbidity diagnoses [89,125,126]. Although the exact mechanism of how EDCs might be causing these complications is unclear, it is becoming more evident that endocrine-disrupting compounds can profoundly affect immune cells, potentially underlying the roots of these conditions.

Important initiators of inflammation are mast cells, which display an increased expression of histamine and leukotriene receptors after BPA exposure [127,128]. Moreover, this compound has been shown to increase mast cell degranulation and the release of pro-inflammatory lipid mediators in vitro [129]. Increased mast cell activation has been associated with increased fibrosis in the kidney after injury [130], and it can lead to the recruitment of other innate immune cells, like macrophages and neutrophils.

BPA has been shown to decrease phagocytosis in various macrophage models, and similar effects have been observed with phthalates and persistent organic pollutants [131,132,133,134]. However, BPA increases the lipopolysaccharide (LPS)-induced release of interleukin (IL)-1β, IL-6, CXCL8/IL-8, and TNF in neutrophils and macrophages, which could contribute to exacerbated inflammation [131,133,134]. Similar patterns have been seen with other EDCs like mono(2-ethylhexyl) phthalate (MEHP), dibutyl phthalate (DBP), and 17α-ethinylestradiol (EE2) [131,133,135,136]. Some authors have contrarily shown that BPA, as well as perfluorooctane sulfonate (PFOS) and phthalates, decrease the production of nitric oxide (NO), IL-1β, IL-6, and inflammatory lipid mediators from macrophages [133,135,137,138]. Stimulation of polarized mouse peritoneal macrophages with BPA promotes inflammatory M1 macrophage functions, such as the secretion of TNF, IL-6, CCL2/MCP-1, and the expression of iNOS and IRF5, while impairing the functions of anti-inflammatory M2-polarized cells [139]. Although M1 macrophages are efficient in clearing infections, their overactivation in tissues like the testes has been associated with increased oxidative stress that can lead to lower fertility [140,141].

Neutrophil extracellular traps (NETs) are an essential anti-microbial function of neutrophils that limit the spread and proliferation of pathogenic bacteria. However, their excessive release and impaired removal have been suggested to ultimately lead to fibrosis and loss of function of the affected tissue over time [142]. In this sense, BPA, di(2-ethylhexyl) phthalate (DEHP), and benzyl butyl phthalate (BBP) increase the release of NETs in murine models of lung and kidney injuries, exacerbating tissue damage [136,142,143]. Additionally, other authors described how BPA exposure can further affect the expression of activation markers in neutrophils, potentially compromising their anti-microbial functions once activated [144]. In this sense, BPA, BPS, and their glucuronidated metabolites can modulate glycolysis and the functional responses of human neutrophils following their activation with the microbial peptide N-formyl-methionyl-leucyl-phenylalanine [92]. Altogether, EDCs can modulate various innate immune functions that might impair the clearance of pathogens while promoting inflammation. Given their role in establishing the environment that will define adaptive immune responses, exposure to EDCs could lead to dysregulated or exacerbated inflammation.

DCs mediate the interplay between innate and adaptive immune compartments, and their activation defines their efficacy in antigenic presentation. In this regard, BPA, PFOS, and diethyl phthalate (DEP) have been shown to decrease the expression of MHC-II and of the costimulatory markers CD80 and CD86 from DCs [145,146,147]. Additionally, exposure of mice to BPA dysregulates gene expression associated with TCR signaling in lymph node T cells [148]. Hence, EDCs could potentially impair the establishment of an efficient memory response. After an infection, populations of T resident memory cells develop and establish themselves in the local tissue, where they become essential for mediating immunity against reinfections [149,150]. Hence, a compromised memory population in the genitourinary tract could cause the individual to have recurrent urinary tract infections.

The balance between memory and effector lymphocytes is necessary to control tissue damage and promote pathogen clearance. BPA-treated mice displayed an increased proportion of CD4^+^ T cells in the thymus [148], which can compromise CD8^+^ T cell proliferation [151]. Similarly, oral exposure to DEHP and MEHP in mice leads to increased CD4^+^ T cells and secretion of IL-4 and IL-13, promoting a Th2 response due to impaired CD8α^+^ DC maturation [152]. The polarization of T cells can further be affected by EDCs. For instance, high doses of orally administered BPA increase the production of Th1 and Th2 cytokines [153], yet lower doses decrease IFN-γ release [151]. Similarly, exposure to BPA in mice before a parasitic infection increased Th2 cytokines such as IL-4, IL-10, and IL-13 [154]. Th2 responses in the bladder are essential to promote urothelial regeneration after exfoliation, yet its predominance may prevent proper clearance of bacterial infections and compromise bladder function after repeated infections [155]. Th2 responses can also downregulate inflammation and antigen presentation by DCs, which can ultimately impair the establishment of a proper memory response [155]. Nevertheless, despite causing this response in T lymphocytes, BPA has also been seen to decrease the release of IL-10 and IL-13 from innate immune cells like circulating monocytes, M2-polarized macrophages, and the human monocytic THP-1 cell line [134,139,146]. Thus, the effects of EDCs on the polarization and outcome of an immune response might depend on the original immune challenge and the immune cells that are present and dominate the response at the time of exposure.

Equally as important, Treg cells are crucial for modulating inflammation and promoting angiogenesis. Exposure of mice to BPA decreased the number of Tregs in splenocytes [154]. Similarly, people who were exposed to DBP prior to triggering allergic responses displayed reduced levels of Tregs in circulating blood and a different chemokine profile [156]. The decreased maturation of Tregs could potentially lead to exacerbated inflammation upon infection in the genitourinary tract while promoting a shift in T cell polarization that could affect the outcome of infections. Furthermore, a low number of Tregs during pregnancy would allow the overactivation of immune cells against the fetus and the risk of miscarriage. Altogether, EDC exposure affects various functions of adaptive immune cells, which could ultimately promote reinfections and disturb the homeostasis and functions of different organs in the genitourinary system.

Overall, it remains clear that EDC exposure can lead to dysregulated immune responses, and the complex interplay between cells can be altered and lead to undesirable conditions (Figure 3). Although it is crucial to keep learning about EDCs and their effects, various challenges remain present. For instance, different models have displayed different behaviors to EDC stimulation. As such, the species (mouse vs. rat), genetic background (BALB/c vs. C57BL/6 mouse strains), source of the cells (cell lines vs. murine cells), sex (male vs. female), and the type of immune cell being studied can all affect the outcome of EDC exposure. Hence, the limitations of their study should always be considered before translating the findings into human physiology. Understanding how these compounds could interact and modulate immune cells may uncover mechanisms underlying diagnoses associated with EDCs, like recurrent infections, exacerbated inflammation, infertility, and cancer.

## 4. Anatomy and Development of the Urogenital System

Sexual reproduction has led to the specialization of certain organs to play specific roles during mating and reproduction, like the stamens and pistils of plants. It should be noted that, sometimes, evolutionary processes initially use similar organs, which will differentiate during embryonic development to generate sexual dimorphism. This differentiation during embryogenesis or later in the individual’s life (for example, puberty in humans) will require close control. The disruption of these phases by endocrine disruptors, for example, can have significant consequences for the individual and potentially for the species if it becomes widespread [157,158,159,160,161].

### 4.1. The Female Genital Apparatus

**Figure 4:**
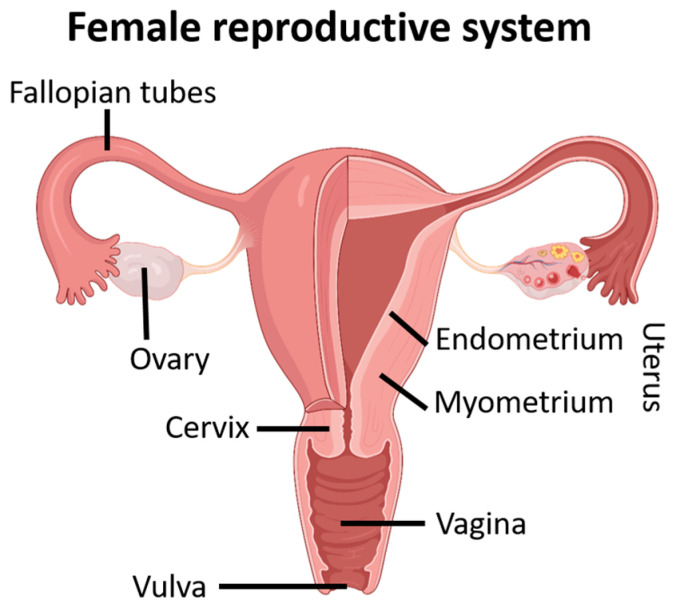
**Schema of the female reproductive system.** The female reproductive system is composed of the ovaries, which produce the oocytes, and the fallopian tubes, which allow the transport of these cells to the uterus, where fecundation takes place. The uterus comprises several structures, including the endometrium and myometrium, to host the embryo and nourish its development. The vagina is the muscular canal that serves during intercourse and delivers the baby during childbirth.

The female genital apparatus is divided into the internal and the external genitalia. The internal female genitalia are composed of the ovaries, fallopian tubes, uterus, cervix, and vagina (Figure 4). These organs allow women to play a complex role in reproduction, as not only does their body need to produce gametes, but it also needs to support the development of an embryo.

#### 4.1.1. The Ovaries

The ovaries are the female gonads, and they play two roles: to produce female gametes (eggs) and to secrete the female sex hormones, i.e., estrogens and progesterone [162]. Estrogens include estrone (E1); estradiol (E2), which is the most abundant hormone; and estriol (E3) [73]. The ovaries are held in the peritoneal cavity by several ligaments and are supplied by the ovarian arteries branching from the abdominal aorta [163]. The external surface of the ovary is surrounded by fibrous albuginea and covered by a layer of cuboidal epithelial cells called the germinal epithelium. The cortex is more profound in the ovaries and contains the gametes, ranging from the immature oocytes to the mature Graafian follicles. Finally, the medulla is located in the center of the ovaries and includes the primary nerves and blood vessels [164]. Each month, women of childbearing age experience ovulation, where one of the mature follicles ejects its oocyte from the ovary to the fallopian tube to become fertilized by a sperm cell.

#### 4.1.2. The Fallopian Tubes

The fallopian tubes, also called uterine tubes, form the initial portion of the female genital tract (Figure 4) [163]. Each tube is characterized by a thinned segment, the isthmus, which opens into the superolateral region of the uterus. The distal portion of each tube enlarges and wraps around the ovary, forming the ampulla, where fertilization usually takes place. The fallopian tubes are covered with a simple prismatic epithelium where ciliated cells can be found. The oocyte can progress towards the uterus due to the smooth muscle layers’ peristalsis and the cilia’s rhythmic beating. The non-ciliated cells of the mucosa have many microvilli and produce a secretion that moistens and nourishes the oocyte [165,166]. Once fertilized, the oocyte is collected by a hollow and muscular organ with thick walls intended to sustain it for approximately nine months: the uterus.

#### 4.1.3. The Uterus

The uterus is found in the pelvis, between the rectum and the base of the bladder [163]. The uterine wall is made of a mucous membrane called the endometrium, which is highly vascularized and rich in glandular cells. Its thickness varies during the menstrual cycle. The middle layer is a smooth muscle called the myometrium. The perimetrium is the outer serous layer of the uterus, which secretes a lubricating fluid that helps to reduce friction [167]. The structure of the uterus narrows at the cervix, which provides the opening from the uterus to the vagina.

#### 4.1.4. The Cervix

The cervix mucosa contains the cervical glands of the uterus that secrete mucus to fill the cervical canal and cover the external opening of the cervix, probably to prevent bacteria in the vagina from entering the uterus [168]. The cervical mucus also blocks the penetration of sperm except in the middle of the menstrual cycle, where its less viscous consistency allows it to pass through the cervix.

#### 4.1.5. The Vagina

The vagina is a thin-walled tube-shaped organ located between the bladder and the rectum that extends from the cervix to the vulva (Figure 4) [169]. The vagina constitutes the organ of copulation in women since it receives the penis and sperm during sexual intercourse. It also allows for delivery during childbirth and the discharge of menstrual flow [170]. The extensible wall of the vagina includes three layers: the adventitia, which is the outer fibroelastic layer; the muscular layer, which is formed of smooth muscle; and the mucous membrane, which is composed of non-keratinized squamous stratified epithelium. Its epithelial cells release significant quantities of glycogen, allowing the resident bacteria to transform it into lactic acid during anaerobic metabolism, leading to the acidic pH of the vagina. This acidity protects the vagina against infections, but it is also harmful to sperm [169,170]. Near the vaginal opening, the mucosa forms an incomplete partition called the hymen that leads to the external genitalia.

#### 4.1.6. The External Genitalia

The external genitalia, or the vulva, comprises the structures mons pubis, labia majora, labia minora, clitoris, vestibule, hymen, Bartholin’s glands, external urethra meatus, and Skene’s gland. The main functions of the external genitalia are to protect the internal genital tract from infection, to assist in micturition, and to act as sensory tissues during sexual intercourse [171,172,173].

### 4.2. The Development of the Female Genital Apparatus

**Figure 5:**
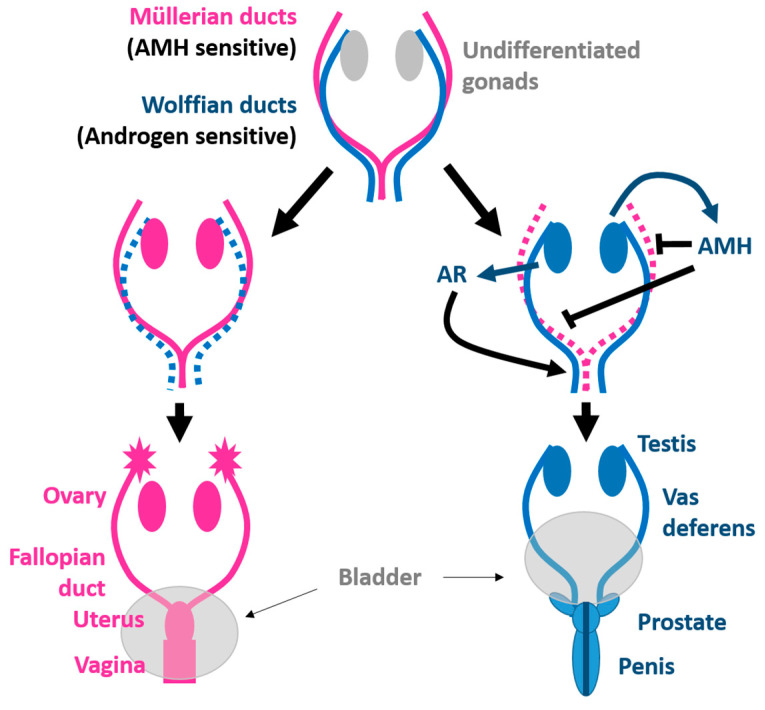
**Development of the genital organs in utero.** Hormones tightly regulate the differentiation of genitalia in men and women. These organs are highly responsive to hormonal signaling from the beginning of their development and throughout adulthood to perform their functions. Female genital organs are represented in pink, whereas male genital organs are depicted in blue. AMH: anti-Mullerian hormone; AR: androgen receptor.

Female gonads initiate their development during the fifth week of gestation. They appear as large groups of mesoderm called genital ridges (or gonadal ridges). The genital ridge initially consists mainly of mesenchyme and cells of underlying mesonephric origin. The paramesonephric ducts (or Müller ducts) develop laterally to the mesonephric ducts (or Wolff ducts) [174,175]. Initially, these two types of canals open into the same cavity called the cloaca [176]. At this stage of embryonic development, the tissue of the gonadal ridges can be transformed into male or female gonads, and it is therefore said to be undifferentiated (Figure 5).

Primordial germ cells migrate to the developing genital ridges shortly after their apparition from another embryo region, probably guided by chemokines [177]. After mitotic proliferation, and depending on the genetic material, the primordial germ cells undergo meiosis and differentiate into mature gametes [177,178]. At this stage, all embryos have a small prominence called the genital tubercle [179]. The urogenital sinus, which develops from a division of the cloaca, is located deep under the tubercle and will grow to form the future urethra and bladder [172,176,180]. The urogenital membrane, which constitutes the external orifice of the urogenital sinus, is located on the underside of the tubercle and is flanked by urogenital folds, surrounded by the labioscrotal tubercles [181].

In the eighth week of development, the cortical part of the immature ovaries forms the follicles. The paramesonephric ducts differentiate to constitute the female genital tract, while the mesonephric ducts, which give rise to the male genital tract, degenerate. The genital tubercle gives rise to the clitoris, and the urogenital membrane becomes the vestibule of the vagina. The urogenital folds and labioscrotal tubercles do not fuse but transform into the labia minora and labia majora. Then, in the absence of testosterone, the female adnexal ducts and the external female genitalia develop [181].

### 4.3. The Male Genital Apparatus

**Figure 6:**
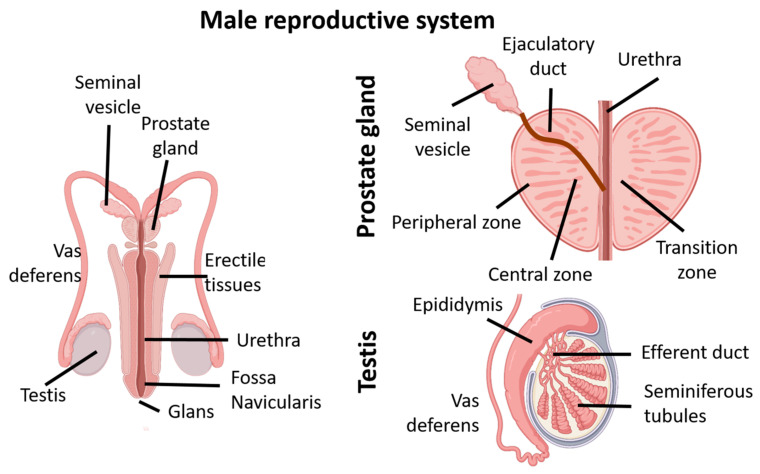
**Schemas of the male reproductive system.** The male reproductive system is depicted in the left panel. More detailed prostate and testis structures are presented in the right panels.

The male genital tract (Figure 6) consists mainly of the testes, the prostate, and the penis. The testes are where germ cells are produced. A tubular structure then allows the transport of these cells to the prostate to form sperm, a liquid rich in various elements necessary for the survival of the sperm until the fertilization of the oocyte. The sperm are expelled through the urethra, which also serves to excrete urine. Urine is produced by the kidneys and transported through the ureters to be stored in the bladder until urination. Although the bladder is not a member of the genital apparatus, it is subject to EDC action and presents histological similarities with the urethra.

#### 4.3.1. The Testes and Vas Deferens

Testes are the male gonads. Humans have two testicles of approximately the same size surrounded by the scrotum, an extension of the abdominal wall. Approximately 80% of the testicular mass consists of tightly coiled seminiferous tubules where spermatogenesis occurs [182]. The tubules are lined with germ cells, which begin to differentiate into sperm cells in puberty [183]. Leydig and Sertoli cells comprise the other 20% of the testicular mass and are required for normal spermatogenesis [184]. Sertoli cells line the tubular walls inside the testes, forming the blood–testes barrier and nursing sperm cells under the control of the follicle-stimulating hormone (FSH) and inhibin [185,186]. They also secrete the fluid that facilitates the transport of sperm cells into the epididymis. Thus, the correct differentiation of Sertoli cells during puberty is essential to spermatogenesis, and it may be targeted by EDCs. Similarly, Leydig cells are the endocrine cells of the testes, as they produce testosterone from cholesterol after the sixth week of gestation [187,188]. The sperm cells produced in the tubules are collected into the efferent ducts until reaching the epididymis, where they are concentrated and continue maturation. Finally, sperm continue to move until reaching a 30 cm-long tube, the vas deferens or sperm duct, where they pass by the prostate.

#### 4.3.2. The Prostate

The prostate is a gland located below the bladder, and it is crossed by the urethra to join the ejaculatory ducts, where the urethral sphincter controls the urine passage from the bladder. The prostatic tissue mainly consists of secretory acini connected by ducts lined by an epithelium, all surrounded by an elastic fibromuscular capsule that allows ejaculation [189,190]. There are three main zones in the prostate: the peripheral zone, which is the largest in volume (70%); the central zone, which surrounds the ejaculatory ducts (20%); and the transition zone around the urethra (10%) [191]. Ultimately, the prostate produces prostatic fluid that participates in the formation of semen, making it alkaline to neutralize the acidity of the vaginal mucosa and promote sperm viability after intercourse [192]. The prostate is an organ susceptible to androgens during embryonic development and throughout the life of the male individual, and estrogen exposure during prostate formation can have harmful effects on its formation and functions [193].

#### 4.3.3. The Penis

The penis is composed of several elements that are required not only for urinary purposes but also for reproductive activities [194]. The urethra is a tubular structure consisting of multiple layers of tissues [194,195]. The structure of the tube is roughly similar to that of the bladder: smooth muscle bundles with intra-fascicular connective tissue, the submucosa, and the urethral epithelium, which can differ depending on the location along the penis [195]. The urethra can be divided into several sections: the posterior urethra consisting of the prostatic and membranous sections and the anterior urethra consisting of the bulbar, pendulous, and fossa navicularis sections [194,196,197]. Like the bladder, the posterior urethra is lined by a transitional epithelium followed by a pseudostratified epithelium. Finally, a squamous epithelium can be found in the fossa navicularis [197]. The urethral epithelia are surrounded by several tissues, including the corpora cavernosa, a highly vascularized tissue that facilitates the retention and drainage of blood during erection [198].

#### 4.3.4. The Bladder

The bladder has no role in the reproductive system; both males and females have this organ. The bladder does not present a sexual dimorphism either, but some molecular differences have been described in a study concerning bladder cancer and EDCs [25]. The bladder is the organ that allows efficient urine storage, and it consists of four distinct layers: the adventitia, the muscular layer, the submucosal layer, and the urothelium [199]. The adventitia is a support layer containing adipose cells. The muscular layer is called the detrusor, and its contraction allows urine expulsion from the bladder during emptying [199]. The submucosa, also known as lamina propria, is a tissue connecting the detrusor and urothelium and is essential to maintaining a well-organized and functional epithelium [200,201]. The urothelium is formed by several layers of cells divided into three types: basal, intermediate, and umbrella cells. Basal cells are mainly cuboidal and undifferentiated progenitor cells. Intermediate cells are partially differentiated cells that are useful for urothelial reparation. Finally, umbrella cells are the most superficial and differentiated urothelial cells. These cells organize on their apical surface a protein complex specific to the urothelium, the uroplakin plaque, which ensures the impermeability of the bladder along with tight junctions between cells [195,199].

### 4.4. The Development of the Male Genital Apparatus

Early in embryogenesis, the reproductive organs of both males and females are initially undifferentiated [180,198]. The male gonad arises from the urogenital ridges, as it differentiates into testicular parenchyma dictated by the Y-chromosome-linked SRY gene, which acts as the master sex determinant directing testes formation [202]. While the gonads are undifferentiated, two duct systems exist in mammals, Wolffian and Müllerian ducts, which eventually develop into male and female ducts [203]. Once the gonad is differentiated, Sertoli and Leydig cells appear [204]. In males, the Müllerian duct degenerates under the influence of the anti-Müllerian hormone secreted by testicular Sertoli cells [205], and the Wolffian duct develops into the adult male reproductive tract under the action of androgens produced by Leydig cells (Figure 5) [206,207,208]. The tubules become lined with germ cells, which begin to differentiate into sperm cells at puberty [205].

The prostate arises from the embryonic urogenital sinus (UGS) through six steps: (i) pre-bud UGS; (ii) the formation of prostatic epithelial buds during the ninth to tenth week of gestation; (iii) bud elongation and branching; (iv) canalization; (v) luminal and basal epithelial cell differentiation; and (vi) secretory cytodifferentiation [191,193,209]. This latter step occurs late in the second or the third trimester of gestation in humans.

Leydig cells are the endocrine cells of the testes, as they make testosterone from cholesterol after the sixth week of gestation. As the UGS develops, it produces a reductase enzyme, which transforms testosterone into dihydrotestosterone, which is ten times more potent than its precursor. In this context, the AR is necessary for prostate induction, as differentiation of both epithelial and mesenchymal compartments requires functional androgen signaling in the epithelium [210,211,212].

The differentiation of male externa” gen’talia spans from the 7th to the 17th week of gestation. In both males and females, the mesodermal mesenchyme migrates to form the genital tubercule, whereas the cloacal membrane develops into urogenital folds [198]. These structures later form the urethra (from urogenital folds) and the penile-surrounding tissues (from genital tubercule) in men, whereas they form the labia minor and the clitoris in women [198]. The urethra tubularizes from the proximal to distal directions [180,213]. At the end of the differentiation process in males, the urethra retains a zone, similar to the vagina, that forms the fossa navicularis [180]. Throughout penile development, androgens play critical roles, as their absence or the modification of their actions generate features like that of the female clitoris [214]. 

## 5. Urogenital Pathologies Related to Endocrine Disruptors

**Figure 7:**
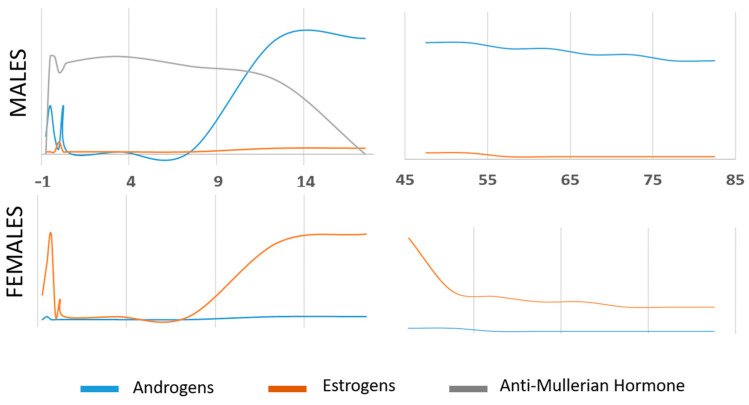
**Sera level of sex hormones during lifetime**. Sera level of sex hormones throughout lifetime is reported in males (upper panel) and females (lower panel). For clarity, the *x–*axis (age) is shared by both panels and was truncated for adulthood before menopause. The level of hormone in the sera is represented as a relative value.

EDCs can affect the development and function of the genitourinary tract and impact reproductive capacity, as a specific hormonal balance influences them during embryogenesis, which later fluctuates depending on the individual’s age [215] (Figure 7).

### 5.1. EDCs and Malformations of the Urogenital Tract

Although multifactorial, many malformations of the male and female urogenital tract and reproductive disorders have been linked to EDCs [216,217]. The effects can be different, and their significance depends on the stage at which the person is affected, going from fetal development to adulthood. Hormone balance is critical to allow proper fetal development, and several clinical studies have linked prenatal exposure to EDCs to an altered development of the urogenital tract.

#### 5.1.1. Malformation of the Female Urogenital Tract

Prenatal exposure to EDCs may be associated with reproductive malformations, adenomas, cysts, and carcinomas in the reproductive tissues. Interference of EDCs with folliculogenesis, steroidogenesis, ovulation, fertilization, and gestation may induce lower fertility, and exposure during early gestation may disrupt intrauterine implantation [218,219]. A study by Swan et al. showed that DES not only increases the risks of developing vaginal cancer in pregnant women but also increases the risk of adverse reproductive outcomes in their daughters exposed to DES during prenatal life [220]. Furthermore, Sato et al. observed an increase in Erα in uterine epithelial cells in a mouse model shortly after a single injection of DES, leading to an estrogen-dependent vaginal proliferation and the appearance of abnormal keratinized epithelial cells [221]. Similarly, although the exposition to a single high dose of BPA led to uterine and vaginal cell proliferation in ovariectomized mice, exposure to much lower yet daily doses of BPA resulted in uterine hypertrophy, hyperplasia, and mucus secretion, along with hyperplasia and cornification of the vaginal epithelium [222,223].

Various groups have consistently observed the epigenetic effects of multiple EDC compounds that lead to altered gene expression. For instance, Li et al. demonstrated in a mouse model that, in utero, DES decreases fertility through epigenetic modifications in the germ cells (sperm and egg cells) forming in exposed fetuses [224]. Similarly, DNA demethylation of CpG/-464 upstream from the lactoferrin promoter was found in the uteruses of mice in specific response to DES treatment in neonatal mice, even for ovariectomized mice [223]. However, this site-specific demethylation typically requires ovarian hormones to occur. As Nelson et al. showed in a mouse model, endocrine disruptors can also impact growth factor expression. Indeed, expression of EGF, which is usually under steroid hormone control, has been induced by neonatal exposure to DES and is persistent even in adult animals [225], effects that other teams have also observed [223,226]. Gene expression of different factors such as TGF-α, insulin-like growth factor (IGF)-I, IGF-II, tumor necrosis factor (TNF), and keratinocyte growth factor (KGF) have also been shown to be altered in neonatal DES-exposed uteruses [223,227].

The link between neonatal exposure to EDCs and polyovular follicles has also been established. Indeed, in DES-treated mice, polyovular follicle incidence was estimated to be 120 to 340 times higher than in the control group [223,228]. Going further, Iguchi et al. succeeded in ovulating polyovular follicles induced by neonatal DES exposure using gonadotropins to compare their fertilizability to non-exposed individuals. In this study, following in vitro insemination, 77% of oocytes from uniovular follicles of control mice developed embryos up to the eight-cell stage. However, only 66% of oocytes from uniovular follicles and 47% of the polyovular follicles of DES-exposed mice developed to the same stage [229]. Similar results have been shown in humans, with higher rates of infertility and anatomical structural defects in women exposed to DES [230].

Exposure to EDCs after birth can also impact the urogenital tract and the reproductive capacity of women. A relationship between EDCs and the disruption of ovarian function has been observed, including lower fertility and fecundity [216], alteration of placental ion transportation, early menopause [87], fibroids, endometriosis [231], modified ovarian cyclicity [232], and altered pubertal timing [233,234]. Indeed, clinical data showed precocious puberty and earlier menarche in girls exposed to persistent organic pollutants [235]. This pubertal alteration was tested in female rat models exposed to parabens, and a significant delay in the date of the vaginal opening, a disruption of the estrous cycle, and a decrease in corpora lutea were observed [236,237]. Therefore, these chemical compounds can impact and dysregulate the ER-dependent transcriptional signaling pathways and induce early or late puberty. Moreover, these effects can be observed with EDC concentrations as low as 1000-fold lower than endogenous estrogen [238].

During adulthood, exposure to EDCs can increase the incidence of hyperplasia of the myometrium, endometriosis, polycystic ovary syndrome, premature ovarian failure, and uterine fibroids in women [239,240,241,242]. Usually, around the age of 50, women will naturally experience genitourinary menopausal syndrome (GMS) [243]. Introduced to the medical community in 2014, GMS is characterized by a diversity of symptoms caused by decreased estrogen production [243]. Symptoms can comprise urinary, genital, or sexual manifestations, all of which can lead to a reduced quality of life of individuals [244,245]. One of the main problems related to GMS is vulvovaginal atrophy, which is present in more than two-thirds of cases [243]. In addition to the thinning of the vaginal wall, there is also a diminution of glycogen concentration, an increase in pH, and a reduction in the presence of *Lactobacilli*, all of which can alter the physiological balance of the vagina.

There is no extensive literature on the effects of EDC exposure and menopause, but various epidemiological and animal studies have suggested that EDCs can accelerate the onset of menopause. For instance, levels of EDCs in bodily fluids can be correlated with decreased follicle counts, dysregulated hormonal balance (mainly between estradiol and FSH), and reduced quality of oocytes [246]. In animal models, exposure to BPA causes the reduction of primordial follicles and promotes their degeneration [247]. Similarly, BPA can alter the balance of various hormones involved in ovulation, increasing the length of diestrus in females [248]. Taken together, exposure to EDCs during multiple periods of life could affect the development and maturation of oocytes as well as hormonal balance later in life, potentially risking an earlier onset of menopause.

Given the risks of providing estrogen as a direct treatment [249,250], various non-hormonal alternatives have been developed for the treatment of GMS [251]. Interestingly, phytoestrogens appear to alleviate multiple symptoms of menopause [252]. Still, their long-term effects remain to be elucidated. Furthermore, selective estrogen receptor modulators (SERMs) or down-regulators (SERDs) are commonly used as non-hormonal therapies. These medications interact with estrogen receptors to either block or promote estrogen signaling in a tissue-dependent manner [253]. Although these drugs are effective in reducing symptoms of estrogen deficiency and cancers associated with low estrogen levels [254], recent studies have highlighted their adverse effects. In this sense, numerous SERMs and SERDs have been shown to modulate the functions of different immune cells in the context of inflammation, often promoting a microenvironment propitious for cancer development [255]. Moreover, these drugs can display different effects depending on the dose to which cells are exposed, further diversifying their potential effects [256]. Although no studies specifically address the effects of other synthetic EDCs on these medications, it could be hypothesized that EDCs could be equally capable of competing with or interfering with SERMs and SERDs, ultimately reducing the efficiency of these treatments given their ability to interact with shared pathways.

#### 5.1.2. Malformation of the Male Urogenital Tract

Like in women, various studies have shown an increased incidence of reproductive anomalies in men after exposure to EDCs, such as genistein and DES, like reduced anogenital distance, hypospadias, cryptorchidism, reduced fertility, oligospermia, and altered testicular function [157,158,216,217,257,258,259,260,261,262,263,264]. Hypospadias result from an interruption in the fusion of the urethral folds during fetal development, which is under hormonal control [180]. Similarly, the descent of the testicles is also known to depend on testosterone, particularly during the inguinal phase, which is the most frequently disturbed phase leading to cryptorchidism [265]. These data have also been supported by studies in rodents showing reproductive dysfunction due to anti-androgenic and estrogenic EDCs [266]. Kim et al. demonstrated the impact of maternal exposure to estrogen or EDCs with estrogenic properties on urogenital development in male offspring, such as a disrupted urethral seam and hypospadias [267]. Mechanistically, the transcription of various genes in urethral tissues was disturbed, including those involved in proliferation, apoptosis, and adhesion signaling pathways, as well as ER, FOXO, and HOX transcription factors, demonstrating the powerful impact of EDCs on urogenital development.

In humans, epidemiological data have indicated that high estrogen levels in the mother during pregnancy are a risk factor for poor semen quality and testicular cancer [265,268]. Additionally, Yoon et al. showed that exposure to EDCs is associated with inappropriate modulation of hormone receptors and can, therefore, alter the development of the male reproductive tract [80]. EDCs might also alter gene expression in males, as clinical and animal data have shown that postnatal exposure significantly decreases sperm quality via genetic and epigenetic activities [269,270]. Consistent with these data, a study by Klip et al. showed that a pregnant woman’s contact with DES exposed three generations to an increased risk of congenital malformations and possibly neurodevelopmental disorders such as Attention Deficit/Hyperactivity Disorder (ADHD) [271]. Another clinical study similarly showed an increased risk of malformations of the genitalia tract in the grandchildren of DES-prescribed women [272]. Transgenerational studies on animals have further confirmed that exposure to EDCs in pregnant mice predisposes the fourth-generation descendants to differential DNA methylation in sperm [273].

EDCs not only interfere during fetal development. Indeed, endocrine signaling is also implicated in childhood, puberty, and adolescence [274]. The link between the exposure of children or adults to EDCs and reproductive disorders has also been reported, with the most common ones being reduced semen quality, infertility, and delayed puberty in boys in contact with persistent organic pollutants [235].

### 5.2. Links Between EDCs and Cancers of the Urogenital Tract

Many cancers exploit sex hormones to their benefit, especially in hormone-sensitive types such as breast, ovarian, and prostate cancer. Breast cancer is extensively studied in relation to EDCs, with some epidemiological studies finding a positive correlation between EDC concentrations in serum or urine and breast cancer risk [275,276]. EDCs can increase tumor incidence and affect cancer progression through various mechanisms, including epigenetic changes and immune modulation [277,278]. Reproductive malignancies are also significantly influenced by hormonal fluctuations, thereby potentially linking them to the effects of EDCs.

#### 5.2.1. Cancers of the Female Reproductive Tract

##### Endometrial Cancer

Endometrial cancer is one of the most diagnosed cancers of the female genital tract [279]. In epidemiologic studies, women with higher urinary concentrations of phytoestrogens had three to four times the odds of developing endometrial cancer [279]. In the case of synthetic EDCs, a case-control study between 2011 and 2014 found higher levels of alkylphenol in the urine of women diagnosed with endometrial cancer compared to healthy controls [280]. Furthermore, nude mice exposed to BPAF exhibit augmented uterine weight, which indicates increased cell growth [281]. Several in vitro studies found that BPA exposure could promote epithelial cell proliferation, either by altering uterine fibroblast growth factor signaling [282], increasing miR-107 expression [283], or promoting ERRγ translocation through EGF-dependent and -independent pathways [284]. Other EDCs, such as DES and the synthetic insecticide fenvalerate, have also played a role in uterine carcinogenesis by impairing nucleotide excision repair capacity [285] or inducing cell cycle progression and collagen expression [241].

##### Ovarian Cancer

Although less common, ovarian cancer ranked as the 14th highest in mortality among all cancers in 2022 [134]. Notably, most ovarian cancers over-express Erα and grow in response to estrogens [286]. Preclinical studies found that EDCs stimulate ovarian cell proliferation either via the ER-CXCL12-CXCL4 axis [286], the GPR30 and IGF1R pathways [287], or the regulation of cell cycle- and apoptosis-related genes [288,289]. In addition, a study demonstrated that phytoestrogens contained in soy can promote the development of granulosa cell tumors (a type of ovarian cancer) in tumor-derived cell lines and mouse models [290]. Consolidating these findings, Park et al. further observed cell growth in ovarian cancer to be stimulated by xenoestrogens through estrogen-dependent pathways [291]. Recent evidence also suggests that small non-coding RNA dysregulation by EDCs might have a transgenerational, cancer-promoting effect on ovarian cancer [292].

##### Cervical Cancer

Despite cervical cancer accounting for approximately 3% of all new cancer cases worldwide [134], its association with EDCs remains understudied and, consequently, not well understood. In utero exposure to DES during pregnancy is hypothesized to increase the risk of developing cervical cancer, although no direct causal link has been demonstrated [293]. In vitro studies, however, showed that BPA exposure disturbed spindle attachment to kinetochore and centriole duplication during mitosis [294] and promoted the migration of cervical cancer cells via the IKK-β/NF-κB pathway in cervical cancer cell lines [295]. Additionally, Zhang et al. found that nonylphenol exposure drives cervical cell malignancy through MAPK-mediated ferroptosis inhibition [296].

#### 5.2.2. Cancers of the Male Reproductive Tract

##### Prostate Cancer

Prostate cancer (PCa) represents a significant health problem for men in developed nations worldwide. It accounts for over 10% of cancer cases in the United States and is the leading cause of cancer deaths in men after lung cancer [297,298,299]. While many instances remain benign and respond well to local therapies such as radical prostatectomies and radiation, around 25% of cases still require systemic treatments [300,301,302,303,304,305]. These high-mortality cases revolve around the ability of prostate cancer to circumvent the need for androgens to proliferate, allowing it to evolve into an ‘‘androgen-independent’’ cancer called castration-resistant prostate cancer (CRPC) [306,307]. CRPC remains the most aggressive type of PCa and accounts for the 30% five-year survival rate in patients with metastatic PCa [308]. PCa initiation and progression heavily rely on the action of the AR, a cytoplasmic transcription factor that regulates several biological pathways implicated in prostate cell growth and survival [309,310,311,312]. This dependence on androgens and their receptor is still present in early PCa cases but drastically changes following hormonal therapies. These therapies target the production of the AR ligands, consisting of an ensemble of hormones such as testosterone and dihydrotestosterone, the latter being the most common and having the most affinity to the AR [313]. Androgen deprivation therapies target hormone production via gonadotropin-releasing hormone agonists or antagonists [314]. Patients undergoing androgen deprivation therapy usually achieve castration levels of circulating testosterone, which leads to a significant reduction in tumor growth but inevitably progresses into CRPC [315].

Many risk factors are associated with PCa initiation and progression, such as genetics, age, diet, and hormonal profile [316]. Recent studies have brought attention to the impact an imbalanced hormonal profile has on multiple hormone-sensitive cancers, such as PCa. These efforts not only look at native hormone production but also at the impact of the abundance of EDCs routinely found in the human body [316]. Whether these EDCs are naturally found in the environment or are manufactured via industrial practices (heavy metals, pesticides), their impact on the endocrine system and hormone-sensitive cancers such as PCa is a real and ever-present concern.

In addition to the AR, the prostate epithelial and stromal cells also possess ERα and ERβ. The ERβ is thought to have tumor-suppressive activities [317,318,319], whereas ERα is associated with oncogenic functions [320,321,322]. The over-activation of ERβ, like the AR, is related to the initiation and progression of prostate cancer [323]. The mutated form of the AR, present in CRPC, can, in some cases, allow the binding of estrogen, which in turn grants additional stimuli for PCa growth [324,325].

Arsenic, for example, is a heavy metal that is classified as a human carcinogen in its inorganic form and also qualifies as an EDC [326]. Its exposure is associated with high PCa risks due to its affinity for the ER and thyroid receptors [327,328]. In humans, high arsenic exposure has led to the transformation of prostate epithelial cells into malignant forms characterized by the inhibition of apoptosis, abnormal DNA methylation, and stromal invasion [329,330]. In other studies, arsenic exposure induced androgen independence in healthy prostate epithelial cells [331]. Cadmium, found in products such as fertilizers and batteries, is another heavy metal that acts as an EDC [332]. Long-term exposure to cadmium produces reactive oxygen species and neoplastic transformation in lung and prostate tissues [333,334]. It has also been reported that cadmium induces high proliferation in prostate cells via activation of the ARs and the increased production of connexin-43, a membrane protein implicated in cell junctions [335,336].

Like other EDCs, BPA is also known to have effects on prostate inflammation [337], and it has been shown to reduce steroidogenic enzyme mRNA, thereby lowering hormone production and affecting prostate biology [338]. Some studies have shown that PCa patients have a higher BPA concentration in their urine, and it was suggested to become an additional biomarker for PCa [339,340]. Studies on human PCa cells and healthy cells have shown that BPA exposure leads to disruptions in centrosome duplication, suggesting cancer initiation and an increased potential for cancer aggressiveness and metastasis [340,341].

Many of the compounds often present in pesticides, herbicides, and insecticides have been suggested to increase the incidence of prostate cancer risk or its aggressiveness [342]. These studies are, however, mostly done on farmers, so the exposure to EDCs is relatively high. For instance, exposure to the insecticide dimethoate, an organophosphate, was correlated with increased PCa risk and deregulation of the endocrine system due to its ability to interact with thyroid receptors [342,343,344]. In an article by Koutros et al., four commonly used pesticides were shown to increase PCa aggressiveness. In contrast, the overall PCa risk only increased for patients with a family history of PCa [345]. In another study, the use of herbicides significantly increased the PCa risk following continuous exposure by farmers, but only for young individuals [346].

EDCs can also have a cumulative effect, as shown by Boberg et al. The authors exposed rats to a mixture of EDCs and showed a significant reduction in prostate weight compared to controls. EDCs also exacerbate hyperplasia in their prostates, a similar effect observed when treating rats with anti-androgens. This EDC mixture was also shown to reduce ER mRNAs, which results in delays in prostate development and differentiation. Finally, the inhibition of ERβ further impacted prostate epithelial proliferation, increasing the risk for PCa [347].

##### Testicular Cancer

Testicular cancer (TC), although quite rare in the general population, remains the most common neoplasm in men between the ages of 15 and 40 [348,349,350]. The main hypothesis nowadays, considering the origin of TC, is based on an *in utero* model. It is thought that testicular germ cell tumors, which encompass over 90% of TC cases, originate from fetal gonocytes that fail to mature into spermatogonia during prenatal development and become malignant during puberty or adulthood [351,352]. TC, among other testicular pathologies, such as cryptorchidism, hypospadias, and poor semen quality, are thought to be caused by the same syndrome: the testicular dysgenesis syndrome (TDS) [353]. The TDS hypothesis proposes that the insufficient action of prenatal androgens or the exposure to EDCs, such as androgen agonists or antagonists, negatively impacts testicular health and increases TC risks [354,355]. Although there is a lack of knowledge of any direct causality between these chemical compounds and TC, some studies suggest that EDCs could disrupt prenatal development and lead to TDS [356]. For example, studies on rats have shown that phthalate exposure in utero led to the development of symptoms closely related to TDS in humans [357]. Furthermore, phthalate exposure was shown to induce Leydig cell toxicity or inhibition via its binding to either androgen receptors, aryl hydrocarbon receptors, or peroxisome proliferator-activated receptors (PPARs) [358,359], potentially leading to a significant impairment of testosterone production. Arsenic has also been associated with smaller testicular size, lowered steroidogenesis [360], and increased apoptosis of Sertoli cells [361].

From an epidemiological standpoint, it has been shown that young males born near regions with high pesticide usage were at higher risk of developing TC [362]. This association was further strengthened by correlating prenatal exposure of mothers to EDCs to the risk of TC in male children during or following puberty and with the blood concentration of EDC in the mother [303,363,364]. Although the mechanisms of action of EDCs and their direct impact on TC risks still need to be resolved, these results do suggest that prenatal and infant exposure to EDCs can have severe repercussions on the health of individuals.

### 5.3. Bladder Cancer

Ranking 12th worldwide by number of new cases in 2020, bladder cancer is one of the most common cancerous malignancies [365]. Furthermore, its high progression and recurrence rates significantly burden healthcare systems worldwide due to treatment costs and intensive surveillance strategies [366]. Although the bladder is not a hormone-dependent tissue, EDCs are commonly found in the urine of most individuals [21], potentially impacting the development of bladder pathologies.

A human study on molecular biomarkers of bladder cancer found increased levels of benzo-a-pyrene and BPA in bladder cancer patients relative to healthy controls [367]. Furthermore, a strong correlation has been observed between aromatic amines and the induction of bladder cancer in dogs and primates, whereas rodents are unresponsive to human bladder carcinogens [368]. As for in vitro studies, Dong et al. found that nitrophenol (PNP), an intermediate of organic compound synthesis and a degradation product of the insecticide parathion, could promote bladder cancer progression. The bladder cancer cell line T24 treated with PNP showed increased proliferation and lower apoptosis levels caused by increased expression of cell cycle regulators PCNA, Cyclin-D1, and COX-2 and decreased expression of the pro-apoptotic gene Bax. PNP exposure also increased cell migration and invasion and reduced cellular adhesion to the ECM. Moreover, PNP exposure led to changes in EMT-related gene expression, such as E-cadherin, N-cadherin, vimentin, snail, and slug. Finally, mRNA expression levels of the cancer-promoting genes HIF-1, IL-1β, VEGFα, and K-Ras were increased, while those of the tumor-suppressor genes PTEN, BRCA, and p53 were decreased in PNP-exposed T24 cells, all possibly associated with PPARγ signaling [369]. Other studies on bisphenols found that BPA, BPS, and their glucuronidated metabolites could affect bladder cancer cell metabolism, as chronic exposure to bisphenols and their metabolites decreased the energy metabolism and properties of healthy urothelial cells while increasing them in bladder cancer cells [370]. Furthermore, nanomolar concentrations of BPA could exacerbate the metabolic switch observed in cancer-associated fibroblasts via an increased glycolytic metabolism [371], while ERα activation could promote bladder cancer cell proliferation [372]. These studies, thus, provide additional evidence of the indirect links between EDCs and bladder cancer progression.

## 6. Endocrine Disruptor Regulations Around the World

Several studies have concluded that certain EDCs may pose partial risks while others present imminent dangers [88,373,374]. These findings justify implementing restrictions on EDCs to reduce or even ban certain compounds when necessary. For example, such measures are crucial to protect vulnerable populations like pregnant women and young children during critical phases of development [375]. Restrictions and regulations relating to EDCs may vary from country to country due to various factors, such as their impact on human health or the environment, and they tend to be more prevalent in industrialized countries.

Various measures have been implemented to mitigate the undesirable effects of EDCs, such as cancer development [25,376,377,378]. In Europe, restrictions primarily target the use of these chemicals, notably bisphenols, in sectors such as biocides, cosmetics, and environmental protection. To reduce risks to public health, the European Food Safety Authority (EFSA) set a tolerable daily intake of body weight per day for BPA at 0.2 ng/kg. It has been demonstrated that 8.2 ng/kg of body weight serves as an identified reference point for causing critical effects [379]. Similarly, countries such as Canada, Brazil, and Sweden follow a comparable approach, especially for materials in contact with food, and even ban bisphenols in baby bottles [380,381,382]. In the United States of America (USA), regulatory oversight of food, drugs, and cosmetics is the responsibility of the Food and Drug Administration (FDA), while pesticides and other commercial chemicals are the responsibility of the Environmental Protection Agency (EPA). This division of regulatory responsibilities in the USA leads to overlapping and divergent approaches to monitoring chemicals that can potentially influence the environment and products intended for human consumption, creating deficits in protection against certain EDCs. While BPA is not explicitly banned in the USA, it is expressly prohibited in baby products [383,384]. As a result of restrictions on bisphenols in plastics and increasing market demand, several substitutes for bisphenols have been introduced, including products containing phthalates.

Phthalates are commonly used chemicals frequently found in materials in contact with food. They have been shown to act as EDCs and can be harmful to human health [48,102,104,376]. In Europe, six phthalates have been banned in children’s toys: di(2-ethylhexyl) phthalate (DEHP), dibutyl phthalate (DBP), benzyl butyl phthalate (BBP), di-isononyl phthalate, di-isodecyl phthalate, and di-n-octyl phthalate [385,386,387]. Additionally, both Europe and the USA impose restrictions on phthalates such as DEHP, DBP, and BBP, stipulating that products containing them, like water or food containers, must not exceed 0.1% of their plastic weight to be allowed on the public market [388,389,390,391]. These regulations are based on the toxicological risks associated with these EDCs, aiming to establish safe limits to protect public health [389,392]. They ensure that companies do not surpass the maximum permissible amount of one particular chemical substance released from the material into food, i.e., specific migration limits [389,390,392]. However, European monitoring activities indicate these limits are often exceeded in materials that encounter food, highlighting inadequate control over phthalate use and insufficient attention to their effects [389]. Despite these restrictions, only a few phthalates, among hundreds of others, are regulated, primarily those that have been extensively studied. Consequently, companies often substitute plasticizers with alternatives that may have adverse effects, potentially failing to protect the general population adequately [393,394,395].

While the widespread presence of EDCs such as bisphenols and phthalates in everyday products raises significant public health concerns, pesticides are another critical issue of interest that directly affects the environment and food. Pesticides are toxic substances intended to affect insects by disrupting their endocrine systems. Consequently, pesticides can also be classified as EDCs due to similarities between the endocrine systems of insects and animals. In Europe, pesticides such as chlorpyrifos and diazinon are banned to protect consumers, health, and the environment [383,396,397,398]. In Canada, pesticides are predominantly used in agriculture, with some restrictions, particularly on organochlorines [105,399,400,401,402,403,404,405]. The USA primarily regulates pesticides based on overall human health risks. However, a significant challenge persists due to inadequate testing and information regarding toxicity, exacerbated by the difficulty in swiftly obtaining long-term data. Consequently, pesticides continue to be widely used across the USA, with only a few municipalities implementing restrictions [400,401,402], despite the alarming findings published [105,403,404,405,406,407].

Two common antibacterial compounds in personal care products, triclosan and triclocarban, have drawn criticism. The FDA in the USA prohibited these ingredients from being used in over-the-counter antiseptic washes in 2016 due to safety concerns and potential environmental consequences. In addition, the European Union has enforced more stringent controls on toothpaste and mouthwash products intended for children and set a maximum of 0.3% on the use of triclosan in cosmetic products. These limitations result from increasing evidence suggesting that they may affect reproductive and developmental health [408,409].

Our current understanding of the effects of EDCs on human body systems is incomplete, exposing us to thousands of EDCs without sufficient strategies or knowledge to minimize exposure and potential health risks. Moreover, there are numerous chemicals that have not yet been classified as EDCs because of insufficient evidence despite having known endocrine activity [383]. Therefore, ongoing research and stringent regulatory measures are crucial to mitigate these risks and protect human health and the environment from the impacts of EDC exposure.

## 7. Conclusions

With the growth of human civilization, the need for easily manufacturable and resistant materials and the development of environmental control methods have become urgent to fulfill the populations’ needs. For instance, food security for the increasing population has been ensured with the use of pesticides and fertilizers that augment food production. Similarly, plastics have become the standard material for popular products, given their durability and malleability. However, most of these products contain poorly understood chemicals that affect human health and the environment.

It has been long known that several of these chemicals pose health hazards to humans and animals, and their persistence in the environment endangers multiple generations. Hormones play a crucial role in the development and function of various organs and cells in the human body, and the disruptions in their balance by these chemicals can lead to comorbidities and reduce physiological fitness. These chemicals can induce epigenetic modifications in germinal cells that could lead to malformations during embryonal development or increased rates of cancers in the progeny. Similarly, endocrine disruptors decrease the quality of semen and ovules, risking implantation and the outcomes of pregnancies. Moreover, these molecules can modulate immune cell functions and competency, predisposing individuals to reinfections, cancers, and chronic inflammatory conditions. Unfortunately, most of these chemicals are poorly regulated, and different populations continue being exposed to them throughout their lifetimes.

As individuals, it is crucial to learn about the presence of EDCs and their daily use to make informed decisions on how to avoid them. For instance, the use of pesticides and fertilizers in agriculture should be made public, and individuals should be encouraged to buy produce from regulated and safe providers. Furthermore, individuals could begin using items made of more sustainable and stable products, such as metal and glass, for daily use. Finally, it is essential to be aware of the components of pharmaceutical and beauty products, among others, that could contain unregulated ingredients. Nevertheless, individuals have limited power to reduce exposure to EDCs, and governments must reinforce regulations and periodic surveillance programs to ensure the populations’ safety. The growing evidence on how EDCs affect human health should become an open topic of discussion. As new products continue to appear in the market, it must become urgent to better regulate the use of these chemicals and to ensure better testing to guarantee their long-term safety.

## Figures and Tables

**Table 1:** EDC classification.

Category	Name	Formula or Element	
Endogenous hormones	Estrogens	Estradiol	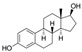
	Androgens	Testosterone	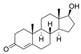
Physical endocrine disruptors	Light	Natural and artificial light	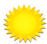 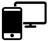
	Temperature	Environmental temperature	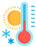
Natural endocrine disruptors (phytoestrogens)	Flavonoids/isoflavonoids	Flavone	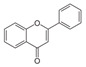
	Pterocarpans	Pterocarpan	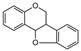
	Enterolignans	Enterodiol	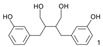
	Coumestans	Coumestrol	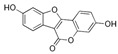
	Mycotoxins	Aflatoxin B_1_	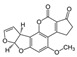
	Stilbenes	Z-stilbene	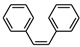
Heavy metals	Chromium	Cr	
	Manganese	Mn	
	Nickel	Ni	
	Copper	Cu	
	Zinc	Zn	
	Arsenic	As	
	Cadmium	Cd	
	Barium	Ba	
	Mercury	Hg	
	Lead	Pb	
Synthetic endocrine disruptors	Phthalates	Diisononyl phthalate	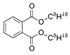
	Bisphenols	BPA	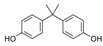
	Pesticides/herbicides/fungicides	DDE	Vinclozolin
		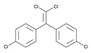	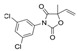
	Pharmaceutical and cosmetic products	DES	Methyl-parabens
		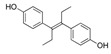	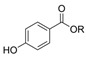
	Persistent environmental contaminants	PCDD	PCB
		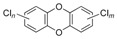	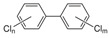

BPA: Bisphenol-A, DDE: dichlorodiphenoxy-dichloroethylene, DES: diethylstilbestrol, PCDD: Polychlorodibenzo-p-dioxine, PCB: Polychlorinated biphenyls.

## Data Availability

No new data were created or analyzed in this study.
